# Morphology-Dependent One- and Two-Photon Absorption Properties in Blue Emitting CsPbBr_3_ Nanocrystals

**DOI:** 10.1021/acs.jpclett.2c00710

**Published:** 2022-05-27

**Authors:** Sol Laura Gutierrez Alvarez, Christina Basse Riel, Mahtab Madani, Mohamed Abdellah, Qian Zhao, Xianshao Zou, Tönu Pullerits, Kaibo Zheng

**Affiliations:** † Department of Chemistry, Technical University of Denmark, Kongens Lyngby 2800, Denmark; ‡ Department of Chemical Physics and NanoLund Chemical Center, Lund University P.O. Box 124, Lund 22100, Sweden

## Abstract

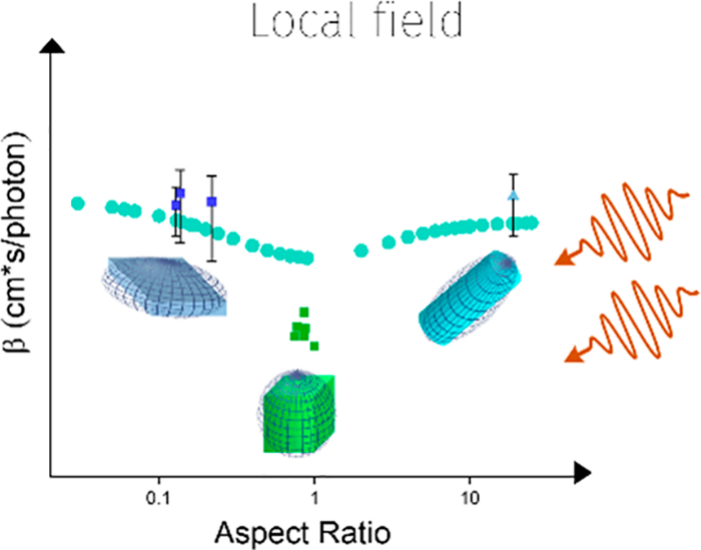

The linear and nonlinear optical parameters and morphologic dependence of CsPbBr_3_ nanocrystals (NCs) are crucial for device engineering. In particular, such information in asymmetric nanocrystals is still insufficient. We characterized the OPLA (σ^1^) and TPA cross sections (σ^2^) of a series CsPbBr_3_ nanocrystals with various aspect ratios (AR) using femtosecond transient absorption spectroscopy (TAS). The σ^1^ presents a linear volume dependence of all the samples, which agrees with the previous behavior in CsPbBr_3_ QDs. However, the σ^2^ values do not exhibit conventional power dependency of the crystal volume but are also modulated by the shape-dependent local field factors. In addition, the local field effect in CsPbBr_3_ NCs is contributed by their asymmetric morphologies and polar ionic lattices, which is more pronounced than in conventional semiconductor NCs. Finally, we revealed that the lifetimes of photogenerated multiexcitonic species of those nanocrystals feature identical morphology independence in both OPLA and TPA.

Colloidal all-inorganic CsPbBr_3_ perovskite nanocrystals (NCs) keep attracting the interest of researchers due to their superior optical properties that benefit their application in solar cells, LEDs, and other optoelectronic devices.^1−4^ Aside from their well-known optical properties in the one linear photon absorption (OPLA) regime, these NCs also present desirable features after two-photon absorptions (TPA). In the TPA regime, the two photons with lower energy than the optical bandgap of the materials are utilized to excite them to an excited state. The use of lower energy photons renders advantageous features in the optoelectronic application, including less phototoxicity, better three-dimensional spatial localization, deeper penetration depth, and lower self-absorption.^1^ The typical applications related to TPA features include nonlinear photonic devices for information and communication technologies,^5^ three-dimensional material microfabrication, information technology, and bioimaging.^6^

The TPA cross section (σ^2^) is the most fundamental parameter to determine the capability of two-photon absorption transitions in the materials. Compared to standard TPA materials such as organic dyes, semiconductor NCs can reach σ^2^ as high as ∼10^6^ GM and higher photostability.^7^ In general, both σ^1^ and σ^2^ exhibit a strong size dependence in semiconductor NCs. Our previous results revealed congruency with literature reports on the linear relationship of σ^1^ to the dimension of CsPbBr_3_ QDs arising from the extension of the density of states.^1,8^ However, most previous studies only focus on the CsPbBr_3_ nanocubes with symmetric dimensionalities. The facile solution synthesis process enables the preparation of perovskite NCs with other morphologies, including 0D quantum dot (QD) structures, 1D nanowires (NW), and 2D nanoplatelets (NPL).^9^ The asymmetry in dimensionality renders them unique superiority in the fundamental research and device applications where optical anisotropy is highly concerned.^10^ In this scenario, whether such dimensional asymmetry influences TPA properties is vital for material and device engineering. Previous reports on CdSe NCs indicated that such an influence exists due to the local field effect on the optical transition of the NCs.^11^ When an object is placed in a vacuum and is exposed to a uniform static electric field, a uniform internal electric field arises, known as the local field. The local field amplitude varies depending on the object’s shape, simplified by the aspect ratio (AR).^12^ Because colloidal NCs are conceptually embedded in a homogeneous nonabsorbing dielectric medium, the classic local field theory applies.^13^ As a result, the TPA cross section is modulated by the AR of the NCs.^11^

This Letter tried to confirm whether such morphologic dependence in the two-photon absorption regime occurs in CsPbBr_3_ perovskite NCs, which usually exhibit less quantum confinement than conventional semiconductor quantum dots. We targeted four NCs featuring the morphology of NPLs and NWs with a wide range of aspect ratios from 0.13 to 19.4. Those NCs present blue emissions due to the low dimensionality instead of the traditional perovskite nanocubes.^14^ We first characterized the one-photon linear absorption (OPLA) and two-photon absorption (TPA) cross section of all the samples extracted from the transient absorption spectroscopic measurement. The same linear relationship of OPLA cross section (σ^1^) to volume was observed consistent with our previous study.^1^ The obtained TPA cross sections (σ^2^) of our NCs ((1.3– 2.3) × 10^6^ GM) are higher than previously reported nanocubes.^1^ However, the TPA coefficients (β) exhibit a typical relationship to the ARs of the samples following the local field theory model observed in other semiconductor QDs.^11^ Moreover, the influence of local field is more pronounced in perovskite NCs, which could be attributed to the additional contribution of polar lattice besides the morphology asymmetry. In addition, we investigated the excited state dynamics of those samples at both OPLA and TPA regimes. No clear morphology dependence of excited state lifetime has been found in both cases. The above results provide a basic overview of linear and nonlinear optical properties of perovskite NCs with various dimensionality, which can serve as guidance for materials engineering and device application.

We synthesized colloidal CsPbBr_3_ nanocrystals (NCs) with different aspect ratios (AR). They are prepared by the hot-injection method described previously in the literature with modifications on temperature and reactant concentration to form nanoplatelets (NPLs) morphology.^15^ To obtain various ARs, different amounts of precursors HBr-OLA (0.8, 1.0, and 1.2 mL) were used for the growth at 90 and 100 °C, as summarized in Table S1. To obtain a more contrasting AR while keeping a comparable dimension of the NCs, a nanowire (NW)-shaped sample was also synthesized. Details of the procedure are presented in the Supporting Information. The samples used for further characterization and spectroscopic studies in the following are all in their original solution form with the concentrations summarized in Table S2. The thickness/diameters of the as-obtained NPLs/NWs range from 1.9 to 3 nm, confirmed by the TEM characterization (Figure 1B). NPLs tend to stack face to face in the TEM images, as illustrated in Figure 1C. Therefore, the top view of the NPLs exhibits a rectangle shape with its dimension representing the lateral length of the NPLs (Figure 1D, top). In contrast, the side view presents parallel stacking of the NPLs where the thickness can be extracted. (Figure 1D, bottom). On the other hand, NWs only exhibit one type of view in the TEM image where both their lengths and diameters are determined (Figure 1D). Table 1 summarizes all the dimensional parameters of the samples.

**Figure 1:**
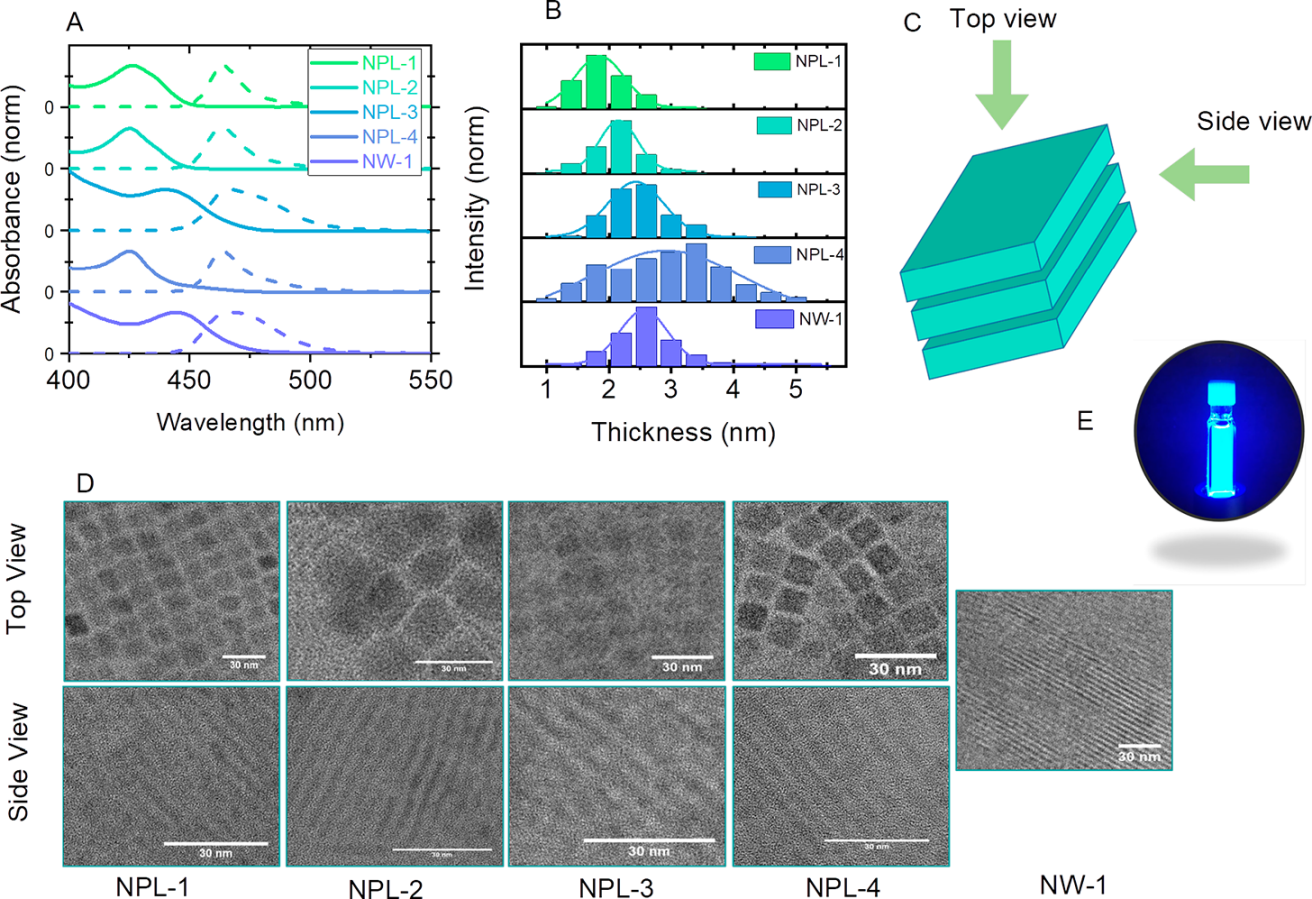
(A) Normalized UV–vis and PL of samples. (B) Histograms of nanoplatelet thicknesses. (C) Diagram of nanoplatelets. (D) TEM top and side view of NCs. (E) Picture of an excited sample with 385 nm light.

**Table 1:** Dimensions of Samples and OPLA and TPA Calculated Cross Sections Compared to Previously Reported Data

sample	shape	thickness (nm)	length (nm)	volume (nm^3^)	AR	σ^1^ (cm^2^)	σ^2^ (GM)
NPL-1	NPL	1.9 ± 0.4	13.9 ± 4.1	367 ± 171	0.14	(4 ± 1) × 10^–14^	(1.98 ± 0.09) × 10^6^
NPL-2	NPL	2.1 ± 0.4	16.3 ± 3.7	557 ± 208	0.13	(5.7 ± 1) × 10^–14^	(2.35 ± 0.07) × 10^6^
NPL-3	NPL	2.4 ± 0.5	11.0 ± 2.7	290 ± 117	0.22	(1.6 ± 1) × 10^–14^	(1.32 ± 0.08) × 10^6^
NPL-4	NPL	2.9 ± 0.9	10.5 ± 2.1	320 ± 134	0.28	(2.2 ± 0.6) × 10^–14^	
NW-1	NW	2.6 ± 0.5	49.5 ± 15.9	334 ± 140	19.04	(2.8 ± 0.9) × 10^–14^	(1.72 ± 0.06) × 10^6^
dSize-QDs^1 ^a	QD	10.5 ± 0.5	12.2 ± 0.5	1345 ± 101	1.16	(12 ± 2) × 10^–14^	(4.5 ± 0.5) × 10^5^
	QD	8.7 ± 0.4	10 ± 0.5	760 ± 64	1.15	(8 ± 1) × 10^–14^	(1.8 ± 0.2) × 10^5^
	QD	6.4 ± 0.4	7.4 ± 0.5	300 ± 40	1.16	(3.1 ± 0.7) × 10^–14^	(0.61 ± 0.04) × 10^5^
	QD	4.5 ± 0.5	5.8 ± 0.6	120 ± 22	1.29	(1.3 ± 0.4) × 10^–14^	(0.29 ± 0.07) × 10^5^
	QD	3.9 ± 0.5	5.2 ± 0.6	80 ± 16	1.33	(9 ± 3) × 10^–15^	(0.16 ± 0.03) × 10^5^
dMorph^8 ^b	QD	6.2	6.2	240.0	1.00	1.87 × 10^–14^	NA
	NPL	3.1	9.8	300.0	0.32	2.68 × 10^–14^	NA
	NW	2.2	36.6	180.0	16.64	1.67 × 10^–14^	NA

a dSize-QDs^1^ results from the study with different size QDs.

b dMorph^8^ results from the study with different shape NCs.

Because the modification on the NC dimensionality will first influence their quantum confinement dominating the electronic structure and optical transition, we first overview the degree of quantum confinement in all those samples. It can be quantified by comparing the critical thickness (*t*) to the Bohr radius of bulk CsPbBr_3_ (*a*_0_ = 7 nm)^16^ as *t*/2*a*_0_ to be between 0.2 and 0.4, which corresponds to the strong quantum confinement regime.^17^ Such a strong quantum confinement also accounts for the blue-shifted exciton absorption and emission bands compared with the bulk phase.^18^ The absorption band edge of the NPLs ranges from 425 to 450 nm, with the corresponding emission peak ranging from 450 to 475 at 385 nm excitation, as shown in Figure 1A. Figure 1E demonstrates the blue emission of the colloidal sample excited with 385 nm CW laser light. We can also confirm the strong quantum confinement by the large exciton binding energy (*E*_b_) calculated from a temperature-dependent PL measurement.^19−21^ The *E*_b_ of NPL-2 is 57 ± 3 meV (see the Supporting Information for more details), which is larger than the value for 8 nm CsPbBr_3_ QDs (40 meV) and within the range of strongly confined CsPbBr_3_ QDs from the theoretical calculation.^16,22^^23^

Next, we extract the OPLA cross section of those quantum-confined NCs via ultrafast transient absorption spectroscopy (TAS) as performed in previously reported studies.^1,8^ All the samples are excited at 400 nm, varying the excitation fluence from 0.2 × 10^13^ to 6 × 10^13^ photons pulse^–1^ cm^–2^. It should be first noticed that the targeted samples are in solution form where the dielectric constant contrast is small between the NCs and solvent compared with air. In addition, the laser illumination induced heating can be neglected at the excitation fluence we used (for a detailed discussion of the photothermal effect, see the Supporting Information). Therefore, the influence of reflective index change or photothermal effect in the absorption cross-section characterization observed in the Z-scan method should be negligible here. The recovery of band-edge ground-state bleach (GSB) represents the evolution of the excited-state population in the NCs. According to the classic assumption, the initial excited exciton population in NCs follows the Poissonian distribution^24,25^
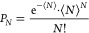
1where ⟨*N*⟩ is the average number of excitons per NCs, *N* is the number of excitons, and *P*_*N*_ is the fraction of NCs with *N* excitons. We can present the average number of excitons per NCs by multiplying absorption cross section σ with excitation intensity *I* as ⟨*N*⟩ = σ·*I*. From eq 1, we can then calculate the fraction of excited NCs, *P*_exc_, as
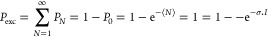
2In reverse, if *P*_exc_ is given, σ can then be calculated from (2). In TA measurement of NCs, the band edge ground-state bleach (GSB) represents the lowest exciton state population after excitation. We can obtain *P*_exc_ by modeling the excitation intensity dependence of the late-time region signal at GSB maximum (*t* > 1 ns), which corresponds to the last remaining exciton population after the Auger process. (A detailed description of the calculation is presented in the Supporting Information.) The analysis provides σ^1^ values of (1.6–5.7) × 10^–14^ cm^2^ for all the samples. The values are within the same order of magnitude as previously reported QDs^1^ characterized with similar methods, as summarized in Table 1.

We implement the fluence-dependent TAS measurement with 800 nm excitation (Figure S5). The TPA cross section σ^2^ can be calculated by relating the one- and two-photon absorption coefficients to experimental fitted parameters *C*_1_ and *C*_2_ in TAS measurement.^1^ The excitation population in the NCs depends on the excitation fluence linearly for the OPLA and quadratically for the TPA (Figure S6)^26^
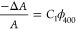
4
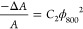
5Here, the ratio between −Δ*A* (i.e., GSB signal amplitude at *t*_0_) and the linear absorbance of NCs (*A*) at exciton transition energy (−Δ*A*/*A*) quantifies the number of photogenerated excitons in QDs within the excitation optical path. ϕ_400_ and ϕ_800_ denote the excitation fluences at 400 and 800 nm. *C*_1_ and *C*_2_ are the fitting parameters from the experimental fluence dependence. The quadratic fluence dependence to −Δ*A*/*A* is direct evidence of simultaneous TPA at 800 nm excitation of our NCs. At the weak excitation-intensity limit (i.e., the average number of excitons per NC ⟨*N*⟩ < 1), the exciton density can be related to the excitation intensity as well as the OPLA coefficient (α^1^) and TPA coefficient (α^2^) as follows:^26^
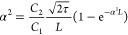
6where τ is the excitation pulse duration (150 fs) and *L* is the sample thickness (1 mm). The TPA cross-section (σ^2^) coefficient can then be obtained from the α^2^ from the following:
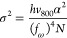
7where *h* is the Planck constant, *v* the frequency at 800 nm, *N* the concentration in particles per cm^3^, and (*f*_ω_)^4^ the local field factor corresponding to the aspect ratio of each sample (for details of the calculation, see the Supporting Information).

The calculated OPLA and TPA cross sections (σ^1^ and σ^2^, respectively) are summarized in Table 1, accompanied by the dimension information about each sample. The relevant results for CsPbBr_3_ NCs in our previous work and literature are also included for a broader comparison. We first study the dependence of the cross sections on the NCs volume and critical dimension (i.e., the length of the smallest dimension, also noted as thickness) as illustrated in Figure 2. The σ^1^ shows a linear relationship with the NC volume as shown in Figure 2A, consistent with previous results,^8^ which indicates the identical optical transition mode to the bulk material.^27^ In addition, the volume-normalized cross-section values (i.e., σ^1^/V) are fixed to be (8.5 ± 0.3) × 10^–17^ cm^2^/nm^3^ independent of the NC thickness, as observed in Figure 2B. The volume independence implies the negligible effect of quantum confinement on the optical absorption transition. We have argued in our previous study that such a linear size dependence of OPLA cross section should still be valid if the excitation energies are far above the band edge (∼400 nm in this case) since the density of states in this region resembled that of the bulk material.^28^

**Figure 2:**
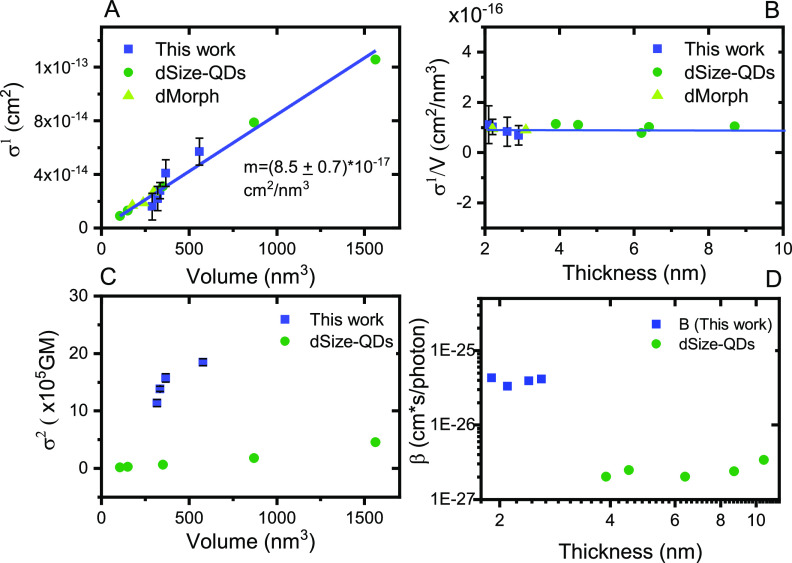
(A) OPLA cross section (σ^1^) at 400 nm vs volume. (B) Volume-normalized OPLA cross-section (σ^1^/V) vs thickness. (C) TPA cross section (σ^2^) at 800 nm vs volume. (D) TPA coefficient β vs thickness. Comparison of calculated results (This work) with reported results from (dSize-QDs)^1^ and (dMorph)^8^.

On the contrary, σ^2^ values of all the NCs exhibit no clear dependence on the volumes of NCs, as seen in Figure 2C. The volume-normalized σ^2^ (also well-known as TPA coefficient β) is also independent of the NC thickness, as shown in Figure 2D:
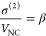
8Previous research demonstrated a simple TPA cross-section power dependence on the NC dimension.^1^ Apparently, such a model cannot interpret the significant deviation among NCs with different morphologies. In other words, there should be other morphological-related parameters that play a critical role in the optical transition.^29^

A previous study of the TPA process in CdSe NCs demonstrates the influence of the local field on the absorption transition when asymmetric dimensionality occurs in the NCs.^11^ This is mainly due to variation in the polarization of the NCs concerning the external electromagnetic field, known as the local field.^11,29^ Such a local field varies depending on the shape of the object.^12^

The local field factor is known as *f*_(*w*)_ for a simplified sphere can be expressed as
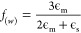
9where ϵ_m_ is the dielectric constant of the surrounding medium and ϵ_s_ is the dielectric constant of the object. Such a model applies very well to QD NCs with symmetric shapes. However, for asymmetric objects like our NPLs and NW, the local permittivity of the object is modulated by a particular shape parameter, the so-called depolarization factor related to the AR. The depolarization factors along the long semiaxis (*L*_*z*_) and perpendicular to the long semiaxis (*L*_*x*,*y*_) of various morphologies have been previously calculated by Osborn.^30^ (see further details in the Supporting Information). Here we can apply a prolate spheroid model to NW and an oblate spheroid model for NPLs.^7,13^

In those spheroids models, the local field can be expressed as a function of depolarization factors (*L*_*i*_ = *L*_*x*_, *L*_*y*_, and *L*_*z*_):^7^
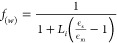
10Now that we have the local field extracted, the TPA coefficient β can then be expressed by the modification of the absorption coefficient of bulk β_bulk_ as shown in eq 11.^31^
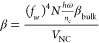
11where *n*_c_ is the density of unit cells in bulk, *N* is the number of cells in the NC, ω is the frequency of the incident radiation, the β_bulk_ value is 3.7 cm/GW CsPbBr_3_,^32^ and *f*_(*w*)_ can be calculated from eq 10. Figure 3 shows the dependence of ARs in β for all the NCs in the study while the dashed line refers to the theoretical modeling based in eqs 10 and 11. The experimental values agree with the basic trend in local field theory model where the lowest β value occurs in the objects with AR close to 1 (i.e., QDs) while our NPLs with AR < 1 and NW with AR > 1 are expected to have higher β values. This is because the symmetric sphere has three identical depolarization factors. However, in ellipsoids that spherical symmetry is broken, causing the polarizabilities to be different in all three dimensions.^12^ Such deviations can significantly alter spectroscopic characteristics.^29^ The pronounced effect we observed in the TPA coefficients is attributed to their quartet dependence of the field factor compared with quadratic dependence for OPLA absorption cross section.^33^ As a result, the TPA absorption efficiency can be significantly enhanced due to local field effects. Such local field effect can also account for the deviation between NCs and bulk materials. As summarized in Table S6, the TPA coefficients in bulk materials are in general higher than the quantum confined symmetric NCs system with negligible local field modulation. However, the strong local field effect in asymmetric systems would enhance the absorption transition that compensates for the loss due to the size effect.
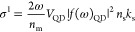
12

**Figure 3:**
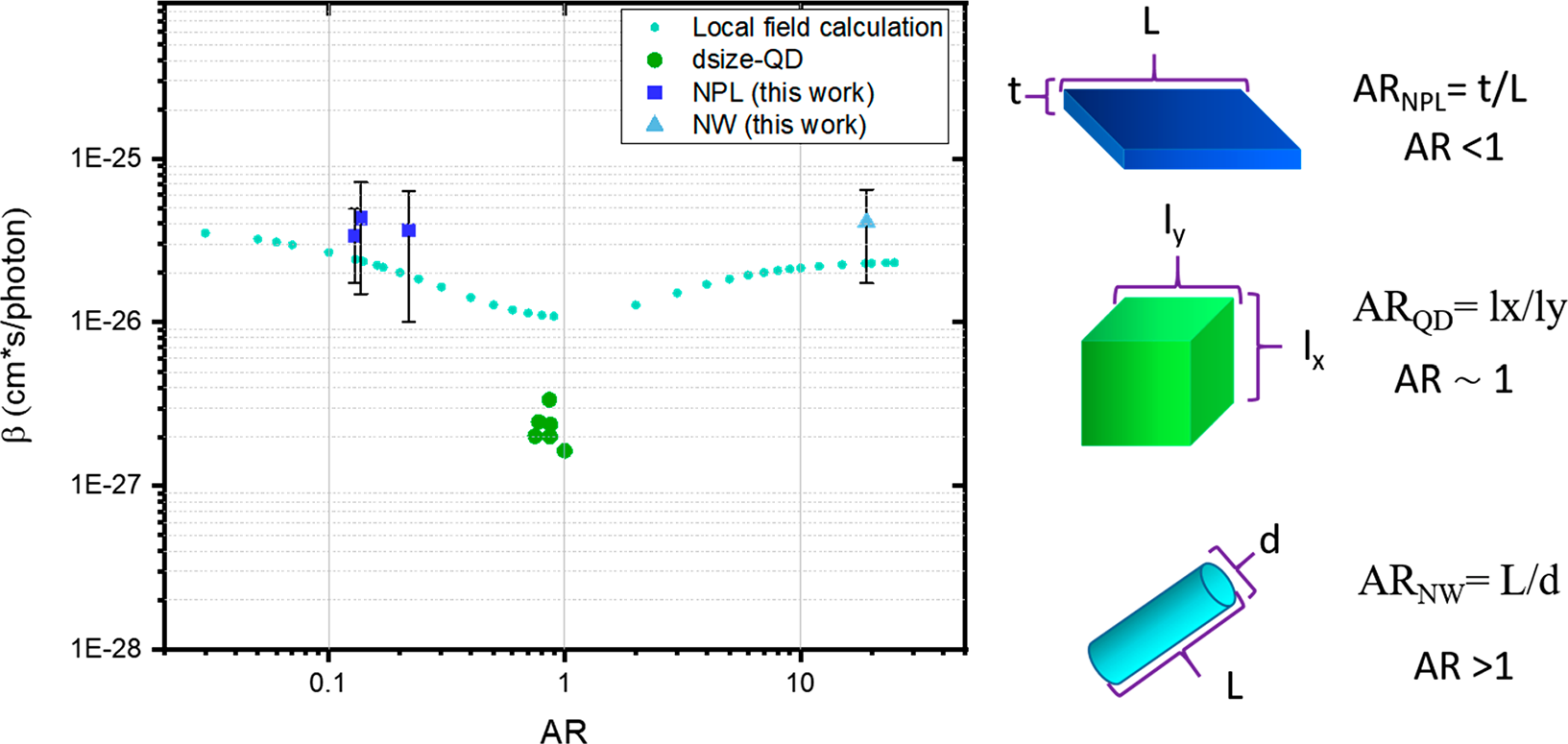
Dependence of TPA coefficient β to AR of different-shaped NCs: blue squares are NPLs, and light blue triangles are NW. The green circles reported values of QDs, and the light green dots are calculated from local field theory at different AR for the different shapes. The AR is defined as AR_NW_ = *L*/*d* for NW, with *L* = length and *d* = diameter, AR_NPL_ = *t*/*L* for NPL with *t* = thickness and *L* = average length, and AR_QD_ = *l*_*x*_/*l*_*y*_ for QD.

However, we noticed the actual aspect ratio dependence in the TPA coefficient values in our CsPbBr_3_ system is more significant than conventional II–VI semiconductor QDs which complied well with the classic local field theory model.^11^ As shown in Figure 3, the deviation of β reaches a factor of 10 between QDs with an aspect ratio of 1 and nanoplatelets with an aspect ratio of 0.15 while the modeling significantly underestimates the deviation.

We believe one of the possible reasons that account for such a discrepancy is the additional local field effect induced by the polarization of the lattice in lead halide perovskite. Compared with II–VI QDs with relative covalent-bond lattices,^34^ lead halide materials exhibit a strong ionic structure with strong polarization. In addition, the preferential lattice orientation was proved along with the growth facets in CsPbBr_3_ nanoplates.^35^ In this scenario, the optical transition dipole moment in CsPbBr_3_ nanoplates cannot be simplified as a point dipole as was done in conventional systems. Instead, the lattice polarization would significantly enhance the local charge inhomogeneity, which is the major origin of the local field effect.^36^ The similar effect has also been reported in organic systems (e.g., fullerene)^36^ or semiconductors (e.g., semiconductor superlattice).^37^

One step further, we studied whether the asymmetric dimensionality would influence the excited-state dynamics of the NCs by characterizing the OPLA and TPA induced exciton (τ^1^) and multiexciton (τ^2^) lifetime, respectively. The exciton lifetime can be extracted from the exponential decay fitting of the GSB decay at the lowest fluence (2 × 10^12^ photons pulse^–1^ cm^–2^) corresponding to the average excitation density ⟨*N*⟩ from 0.04 to 0.10, where no multiexciton recombination can exist (see Figure S8).

The exciton lifetime τ_1_ is shown in Figure 4C with various NCs thicknesses. The multiexciton lifetime can be calculated from GSB decays with higher excitation intensity.^24^ We first subtract the GSB kinetics at high fluence (2 > *N* > 1) with one exciton GSB decay from low fluence measurements (*N* ≪ 1)) with their amplitude normalized at the long time delay >200 ps (Figure 4A). As shown in Figure 4B, the residual decays feature a multiexciton recombination process.

**Figure 4:**
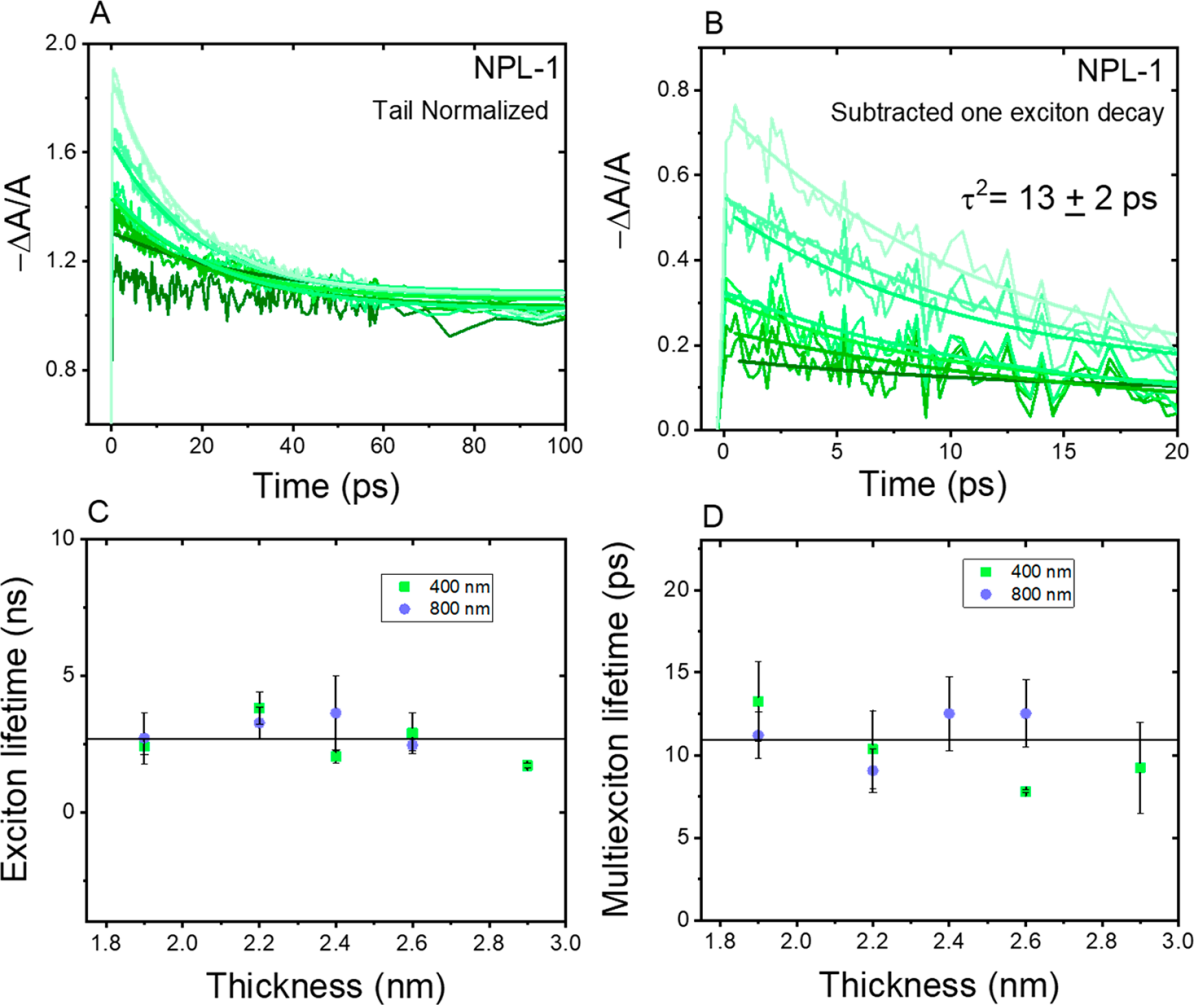
(A) Tail normalized GSB decay for NPL-1 excited at 400 nm. (B) Subtraction of one exciton decay from tail normalized GSB decay for NPL-1 excited at 400 nm. (C) Exciton lifetime at 400 nm excitation and 800 nm excitation in different thickness NCs. (D) Multiexciton lifetime at 400 nm excitation and 800 nm excitation in different thickness NCs.

To distinguish multiexcitonic species, we can analyze the excitation fluence-dependent amplitude of the TA bleach decay components using the model taking into account the Poisson distribution for exciton ⟨*N*⟩^1^ and biexciton ⟨*N*⟩^2^ (details are provided in the Supporting Information).^38^ From the analysis of Figure S9, we conclude that NPL-1 and NPL-3 have a clear biexciton behavior while NPL-4 charged exciton is dominant probably due to the existence of long-lived trap states to extract the photogenerated electrons or holes and the residual species serve as background charges. In NW and NPL-2, species like triexcitons contributed to even higher order of recombination.

The multiexciton lifetime (τ^2^) can then be confirmed as the average lifetime from the monoexponential fitting of all the kinetics at various fluences measured for one sample. Such a strategy has been applied in the analysis of our samples for both TPA and OPLA conditions. As summarized in Figure 4D, the multiexciton lifetimes for all samples are identical between OPLA and TPA conditions, about 5 times longer than the single exciton lifetime. The results obtained for multiexciton lifetime agree with a 9 ± 1 ps biexciton lifetime obtained by previously reported CsPbBr_3_ NPL with dimensions of 4 ± 2 nm thickness and 23 ± 7 nm length.^39^

In Figures 4C,D, it is clear that both τ^1^ and τ^2^ are independent of NC morphology and TPA/OPLA conditions. The identical excited-state dynamics between OPLA and TPA conditions should be attributed to the narrow size distribution of the NCs and especially the very similar and narrow thickness distribution. Our previous study shows that the deviation between OPLA- and TPA-induced excited-state lifetime is mainly induced by the change of excitation distribution among various sized QDs between the two cases.^40^ The morphology- or size-dependent exciton or biexciton dynamics have been widely observed in nanostructures following a universal volume scaling law.^41−43^ However, such volume scaling law is actually integrated with three factors of confinement-induced state mixing, carrier–carrier Coulomb coupling, and surface effects, where each follows a 1/*R* dependence in an ideal symmetric strong quantum confined system (e.g., QDs). In our perovskite NCs system, first, the large NC volume (i.e., 300–500 nm^3^) would still result in a relatively weaker quantum confinement than 3D quantum confined systems and consequently a breaking down of V-scaling.^44,45^ Second, the morphologies of our NCs may influence the carrier–carrier Coulomb coupling via the local charge inhomogeneity and surface effects via the surface-to-volume ratio. Those influences can probably compensate for the confinement-induced effect. These could explain the weak V-scaling and overall morphologic dependence on the multiexciton dynamics.

We have successfully synthesized low-dimensional CsPbBr_3_ blue-emitting NPL and NW with various aspect ratios. The TPA and OPLA cross sections have been characterized from the TAS study. The results show that the σ^1^ shows a linear dependency to the volume of all the NCs as expected. However, their TPA cross sections are dependent on the NC aspect ratios following the local field theory model, where QDs with AR = 1 exhibit the lowest values. Furthermore, we find the local filed effect is more pronounced in our perovskite system than in conventional semiconductor NCs due to the dual contribution of asymmetric morphologies and a polar lattice. Nevertheless, we demonstrated that the excited-state lifetimes of all the NCs are independent of the morphologic at both OPLA and TPA conditions, which can be due to weak quantum confinement and the trade-off between quantum confinement and morphological effect on the multiexciton dynamics. Our findings provide clear guidance for materials engineering in TPA-related applications: Nanostructures with high aspect ratios such as NW and NPLs potentials perform better due to the higher absorption cross section and identical excited-state dynamics.

## Supporting Information Available

Sample preparation, TEM characterization, transient absorption method, temperature-dependent PL, calculation of exciton binding energy, OPLA cross section, TPA coefficient, TPA cross section, local field, multiexciton lifetime, photothermal effect discussion, and comparison of β for different CsPbBr_3_ by the Z-scan method (PDF)
