# Supplementary material for: Morphology-Dependent One- and Two-Photon Absorption Properties in Blue Emitting CsPbBr3 Nanocrystals
Source: J Phys Chem Lett. 2022 May 27;13(22):4897–904. doi: 10.1021/acs.jpclett.2c00710 (PMC9189923; doi:10.1021/acs.jpclett.2c00710)
Supplement: Supplementary file 1 — jz2c00710_si_001.pdf [file jz2c00710_si_001.pdf]

Morphology Dependent One-Photon And Two-Photon Absorption Properties In Blue Emitting $CsPbBr_3$ Nanocrystals

Sol Laura Gutierrez Alvarez[1], *Christina Basse Riel*[l], *Mahtab Madani*[1], *Mohamed Abdellah*[2,3], *Qian Zhao*[1], *Xianshao Zou*[1], *Tönu Pullerits*[2], *Kaibo Zheng*[1,2*]

[1] Department of Chemistry, Technical University of Denmark, Kongens Lyngby, 2800 Denmark.

[2] Department of Chemical Physics and NanoLund Chemical Center, Lund University P.O. Box 124, Lund, 22100 Sweden.

[3] Department of Chemistry, Qena Faculty of Science, South Valley University, Qena, 83523, Egypt.

Corresponding Author

* Kaibo Zheng: E-mail: kzheng@kemi.dtu.dk, kaibo.zheng@chemphys.lu.se

Contents

(S1) Sample preparation... S3

Synthesis Method:..S3

Purification of nanoparticles:...S4

(S2) TEM Characterization S5

(S3) Temperature dependent PL and calculation of exciton binding energy. S7

(S4) Transient absorption (TA).. S7

(S5) OPLA Cross-section calculation S8

(S6) TPA coefficient calculation S11

(S7) TPA Cross-section calculation.. S15

(S8) Local field Calculation.. S15

(S9) Exciton and multiexciton lifetime, multiexciton multiplicity...................... S17

(S10) Multiexciton multiplicity. S18

(S11) Photo thermal effect discussion S19

(S12) Comparison of β for different $CsPbBr_3$ by Z-scan method S21

(S1) Sample preparation

Synthesis Method:

$CsPbBr_3$ NPs of different morphologies are synthesized following a variation of the previously published procedure.[1]

Materials: $PbBr_2$, Cs_2CO_3, Oleic Acid (OA 90%), Oleylamine (OLA 70%), 1-Octadecene (ODE for synthesis), HBr (48%), and Methyl Acetate (for synthesis) were purchased from Sigma Aldrich. Hexane (GC grade) was purchased from Supelco. ODE, HBr, and Hexane are dehydrated for 24 hours using molecular sieves and degassed at 120°C before any reaction. OA and OLA are degassed at 120°C before any reaction.

Cesium oleate synthesis: In a 50 mL pear-shaped flask, 0.163 g of Cs_2CO_3 was added with 9 mL ODE and 1 mL of OA. This mixture is degassed for 1 hour at 120°C. Then it is set under argon for 10 min at 150°C. Afterward, it is preheated to 100°C for nanoparticle synthesis.

HBr-OLA synthesis: In a 100 mL round bottom flask, 1 mL HBr was added with 10 mL OLA. This mixture is degassed for 1 hour at 120°C. Then it is set under argon for 1 hour at 150°C. This is preheated to 80°C for nanoparticle synthesis.

Synthesis of nanoparticles: In a 100 mL three-neck round bottom flask, add 0.14 g of $PbBr_2$, 20 mL ODE, and degas under vacuum for 1 hour at 120°C. Then set under inert conditions (Ar) afterward, and 1 mL of OLA and 1 mL OA are injected. HBr-OLA is injected (0.8 mL or 1.0 mL for NPLs, 1.2 mL for NW). The mixture reached to injection temperature (90°C or 100°C). When the temperature is reached, 0.8 mL of cesium oleate is added swiftly, and the flask is immediately put in an ice bath.

Purification of nanoparticles:

When the reaction mixture is below 50°C, it is centrifuged 15 min 6500 rpm, and the supernatant is collected and precipitated within a mixture 2:1 methyl acetate: supernatant. This is centrifuged for 15 min at 6500 rpm. An excess of methyl acetate is added to the supernatant and is centrifuged for 20 min at 10000 rpm. The precipitate is dissolved in 6 mL hexane.

Table SI. Sample synthesis conditions and TEM characterization.

Name	Injection temperature (°C)	Amount HBr-OLA (mL)	Thickness (nm)	FWHM thickness	Length (nm)	FWHM length
NPL-1	90	0.8	1.9 ± 0.4	0.9	13.9 ± 4.1	4.4
NPL-2	90	1.0	2.1 ± 0.4	0.7	16.3 ± 3.7	6.6
NPL-3	100	1.0	2.4 ± 0.5	1.0	11.0 ± 2.7	3.1
NPL-4	90	1.0	2.9 ± 0.9	2.7	10.5 ± 2.1	4.1
NW-1	90	1.2	2.6 ± 0.5	0.9	49.5 ± 15.9	34.5

(S2) TEM Characterization

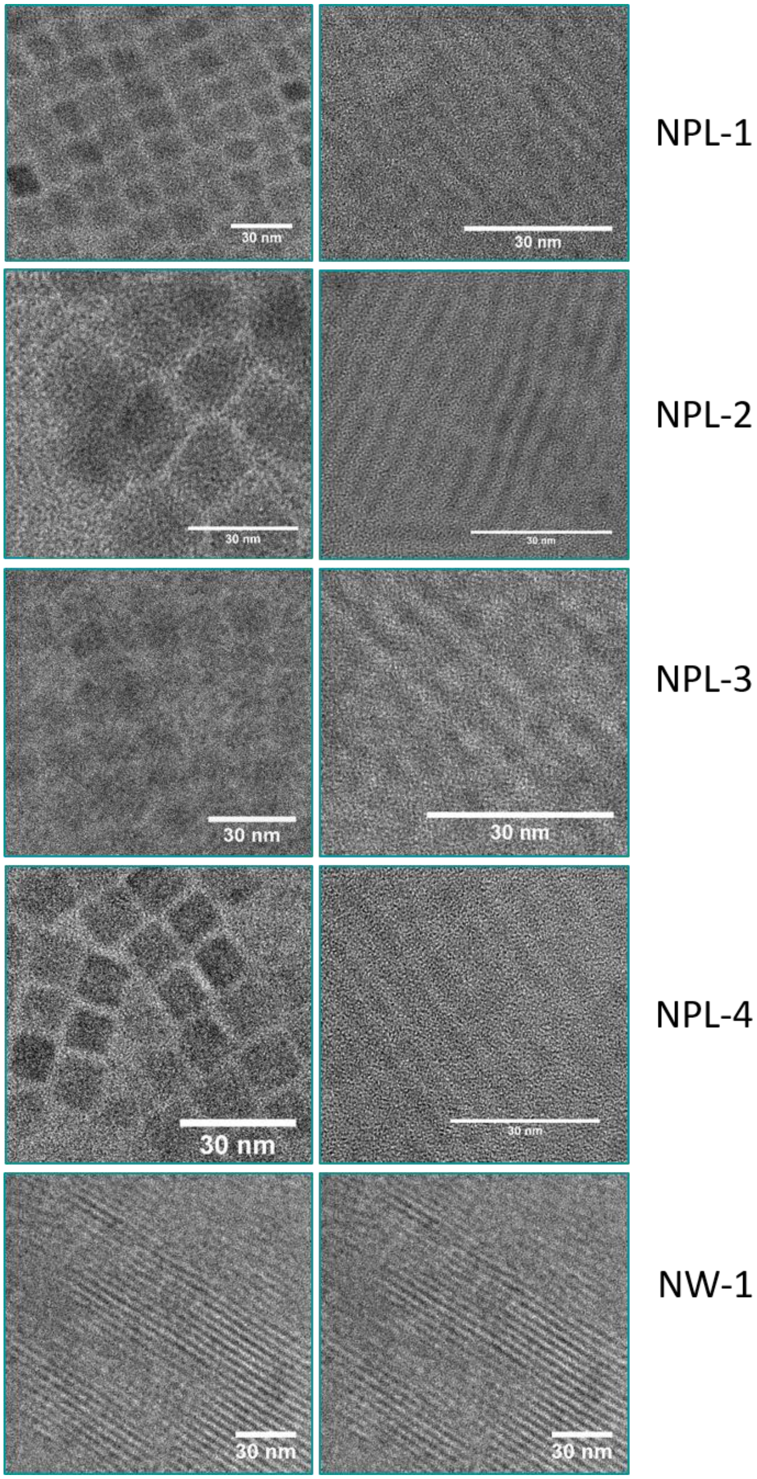

Figure S1 TEM images of samples previously presented left top view right side view.

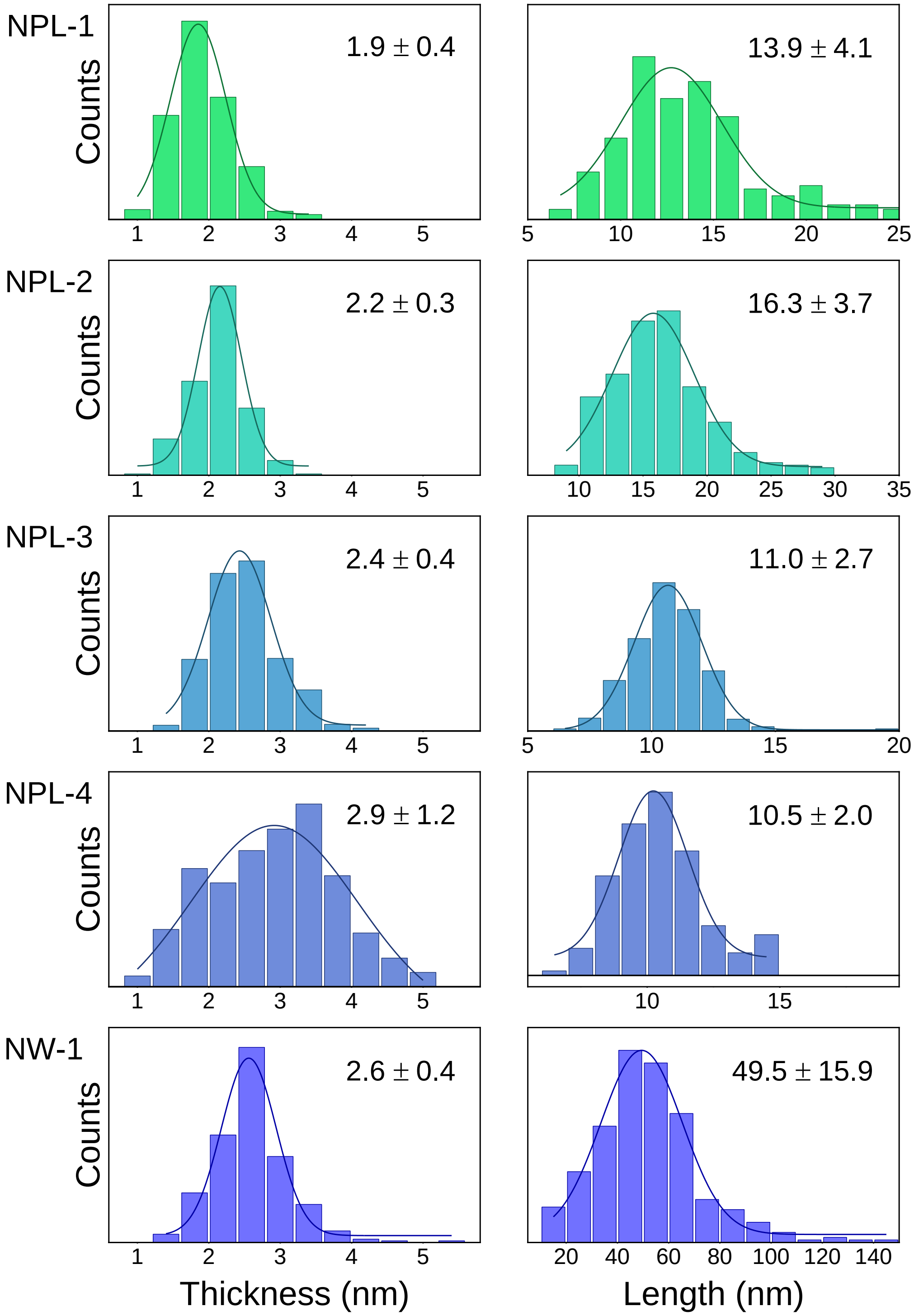

Figure S2 TEM histograms of samples

(S3) Temperature dependent PL and calculation of exciton binding energy.

We calculated the exciton binding energy of our NCs samples using temperature-dependent photoluminescence. PL spectra of NCs were measured at temperatures ranging from 78~300K in a cryostat, and the integrated PL intensities I(T) were calculated. Here the PL intensity decreased with increased temperatures due to the thermal dissociation of excitons at higher temperatures, see fig 3SA. The temperature-dependent PL intensity can then be expressed as follows [2,3]:

$$I(T) = \frac{I_0}{1+Ae^{(\frac{-E_b}{k_B T})}} \quad \text{(S1)}$$

I_0 is the PL intensity at low temperature, and k_B is the Boltzmann constant. From the linear fitting of Ln(I_0/I(T)-1) vs. 1/k_BT plot in Fig. S3B, we can obtain the Eb as the slope of 57 ± 3 meV.

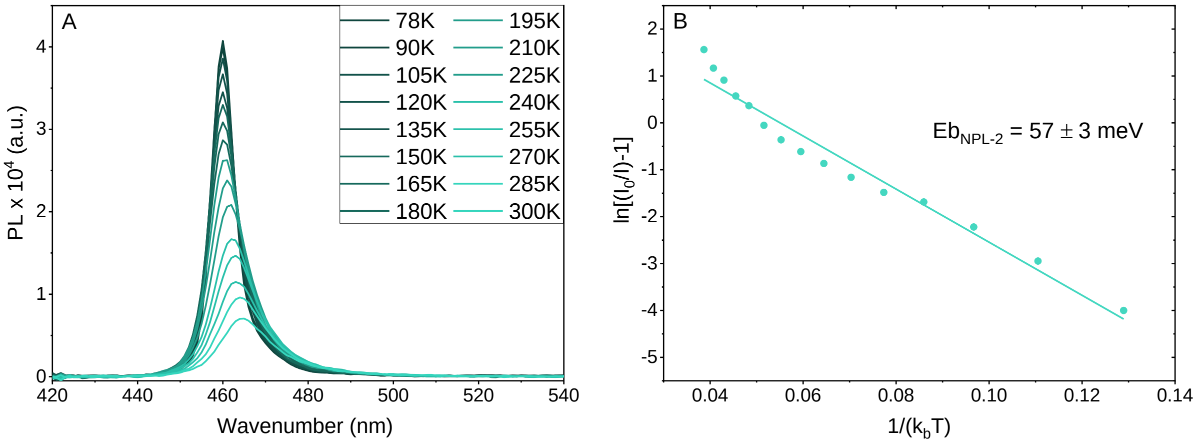

Figure S3 A) Temperature-dependent PL of NPL-2 sample B) ln(I_0/I(T)-1) vs. 1/k_BT plot of temperature-dependent photoluminescence of NCs at temperatures from 78-300 K.

(S4) Transient absorption (TA)

TA experiments were performed by using a femtosecond pump–probe setup. Laser pulses (800 nm, 150 fs pulse length, 3 kHz repetition rate) were generated by Ti:sapphire amplifier with

integrated oscillator and pump lasers (Libra LHE, Coherent Inc.) and Transient Absorption Spectrometer (TAS, Newport Corp.). Briefly, the output of a Ti:sapphire amplifier with integrated oscillator and pump lasers (800 nm, 150 fs, 3 kHz, Libra LHE, Coherent Inc.) was split into two beams which were used to generate 400 nm light through the doubling crystal as a pump beam and to generate the white light through CaF_2 crystal as a probe. The two-photon absorption experiments obtained the 800 nm pump pulses directly from the amplifier. The probe beam was split into two beams, one through the sample and another as a reference. The generated super-continuum was then focused onto the sample and overlapped with the pump beam. The transient spectra were detected with a fiber-coupled CCD-based monochromator (Oriel, Newport). Samples for transient absorption experiments were kept in the dark between each measurement.

(S5) OPLA Cross-section calculation

The OPLA cross-section can be calculated from a pump-probe experiment previously reported. [4,5] The samples are excited with a 400 nm pump with fluence ranging from (0.2 to 6)x10^{13} photons x pulse^{-1} cm^{-2} and probed by white light through CaF_2 crystal. The GSB decay of the NCs represents the exciton population decay in the NCs. Assuming a behavior of the exciton population as a Poissonian distribution[6,7] we obtain:

$$P_N = \frac{e^{-\langle N \rangle}\langle N \rangle^N}{N!} \quad \text{(S2)}$$

where P_N is the fraction of NCs with N excitons, N denotes the number of excitons, and $\langle N \rangle$ the average exciton number per NC. When the optical density of the sample is low, the excitation intensity does not vary in the sample volume, and $\langle N \rangle$ can be expressed as:

$$\langle N \rangle = \sigma^1 * I \quad \text{(S3)}$$

where I is the pump intensity corresponding to the excitation fluence (photons per pulse per excitation area) and σ^1 is the OPLA cross-section at a specific excitation wavelength (400 nm). The method combines equations (1) and (2), and the fraction of excited NCs (P_{exc}) can be calculated as follows:

$$P_{exc} = \sum_{N=1}^{\infty} P_N = 1 - P_0 = 1 - e^{-\langle N \rangle} = 1 - e^{-\sigma^1 I} \quad \text{(S4)}$$

To avoid the effect of multiple excitation decay, the pump intensity dependence is measured from 1 ns signal up to 8 ns. Multiple excitations in a NC decays through the Auger process, which is significantly faster than the radiative decay of the single excitation. This means that once the Auger process is completed, the signal amplitude is proportional to P_{exc}. All excited CsPbBr3 NCs contain only a single exciton at a long timescale. In this scenario, ΔA (I, t≥1 ns) is proportional to P_{exc} and decays mono-exponentially. We can rescale it to the corresponding signal at t=0, named $\Delta A_0(I)$. ΔA_{0max} represents the highest single exciton signal (all NPLs excited) rescaled to t=0.

$$\Delta A_0(I) = \frac{\Delta A(I, t \geq 1\ ns)}{e^{-t/\tau}} = \Delta A_{0,max}(1 - e^{-(\frac{I}{I_0})\langle N \rangle_0} \quad \text{(S5)}$$

Here the lowest pump intensity (lowest fluence) utilized in TA measurement was defined as I_0. We used an intensity low enough that multiple excitons in one NC can be neglected. Clearly, $\langle N \rangle_0$ can be obtained from the performed exponential fit of the $\Delta A_0(I)$ vs. (I/I_0) plots as described in equation (S5), and σ^1 can be calculated according to equation (S3). Figure S4 shows the GSB decays with ascending pump intensity for each NC and in the insert the exponential relationship of $\Delta A_0(I)$ vs. (I/I_0) with the result for each σ^1. In table S2, the values of σ^1 are summarized and the concentration and extinction coefficient.

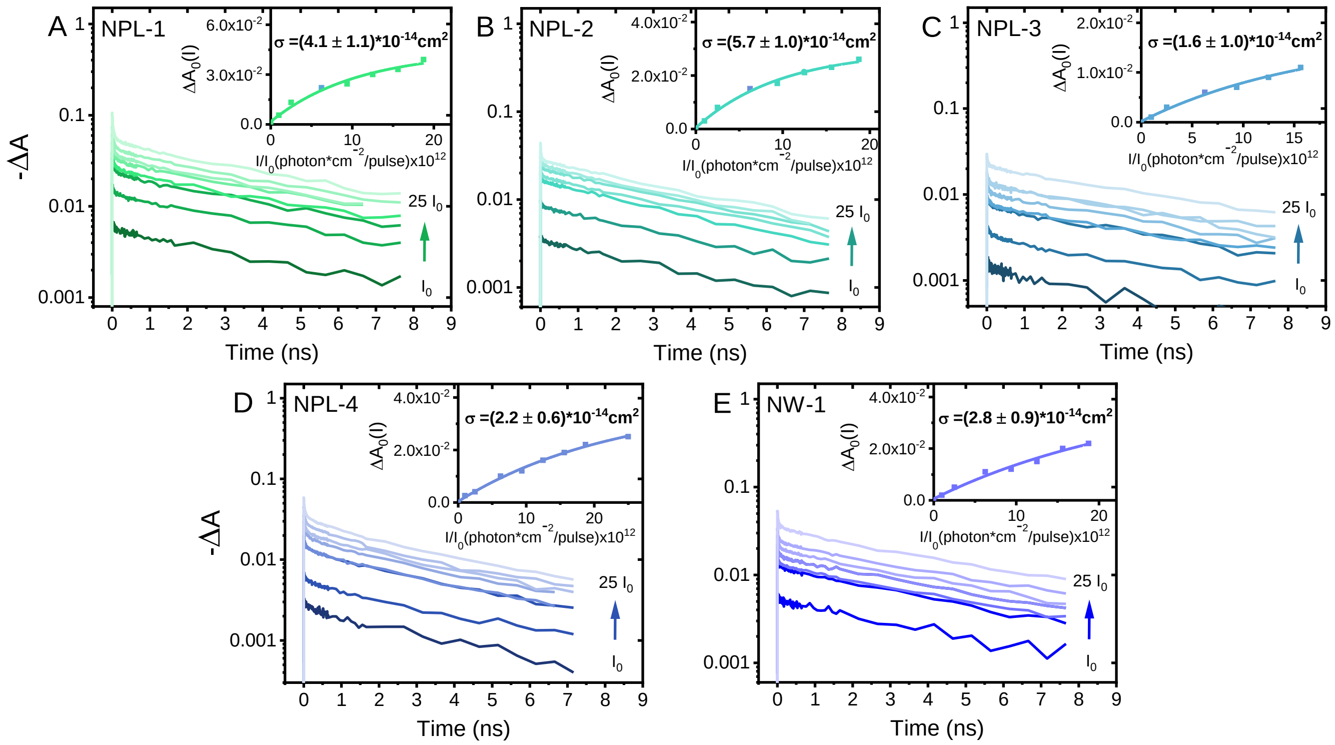

Figure S4. Pump-intensity dependence of TA dynamics for $CsPbBr_3$ NPLs, and NW, excited at 400 nm. With the calculation of absorption cross-section from OPLA.

The extinction coefficient can be calculated from the following relationship

$$\sigma(in\ cm^2) = \frac{1000 \cdot \ln(10)}{N_a} \cdot \varepsilon = 3.825 \cdot 10^{-21} \varepsilon \left(in\ \frac{L}{mol \cdot cm}\right) \quad (S6)$$

And the

Table S2 OPLA cross-section, concentration, and extinction coefficient.

Sample	σ^1 (cm^2)	$\varepsilon_{400\ nm}$ ($L\ cm^{-1} mol^{-1}$)	C (μM/L)
NPL-1	$(4.1 \pm 1.1)x10^{-14}$	$(1.1 \pm 0.3)\ x10^{7}$	1.1 ± 0.3
NPL-2	$(5.7 \pm 1.0)x10^{-14}$	$(1.5 \pm 0.3)\ x10^{7}$	0.6 ± 0.1
NPL-3	$(1.6 \pm 1.0)x10^{-14}$	$(4.1 \pm 0.1)\ x10^{6}$	3.4 ± 0.9
NPL-4	$(2.2 \pm 0.6)x10^{-14}$	$(5.7 \pm 0.1)\ x10^{6}$	1.5 ± 0.4
NW-1	$(2.8 \pm 0.9)x10^{-14}$	$(7.3 \pm 2.3)\ x10^{6}$	1.9 ± 0.6

(S6) TPA coefficient calculation

The two-photon absorption cross-section (σ^2) is calculated from the TAS method, reported previously.[4, 8] The TAS is performed in the samples with an excitation pump of 800 nm, the fluence of the excitation pulse is performed in an increasing manner from (4.3 to 38.5)x10^{14} photons*pulse^{-1} cm^{-2} (Figure S5). The extrapolated amplitude at t=0 from the long timescale of each GSB decay is plotted against the fluence at 800 nm excitation and the 400 nm excitation performed previously for the OPLA. The ratio between extrapolated GSB signal amplitude −ΔA and the linear absorbance of QDs (A) at exciton transition energy (−ΔA/A) quantifies the number of photo-generated excitons in QDs within the excitation optical path. The signal amplitude is proportional to the exciton population in the QDs. The population depends on the excitation fluence linearly for the OPLA (Figure S6, left) and quadratically for the TPA (Figure S6, right) [9]:

$$\frac{-\Delta A}{A} = C_1 * \emptyset_{400} \quad \text{(S7)}$$

$$\frac{-\Delta A}{A} = C_2 * \emptyset_{800}^{2} \quad \text{(S8)}$$

Here, C1 and C2 are the fitting parameters from the experimental fluence dependence reported in Figure 6 and summarized in Table S3.

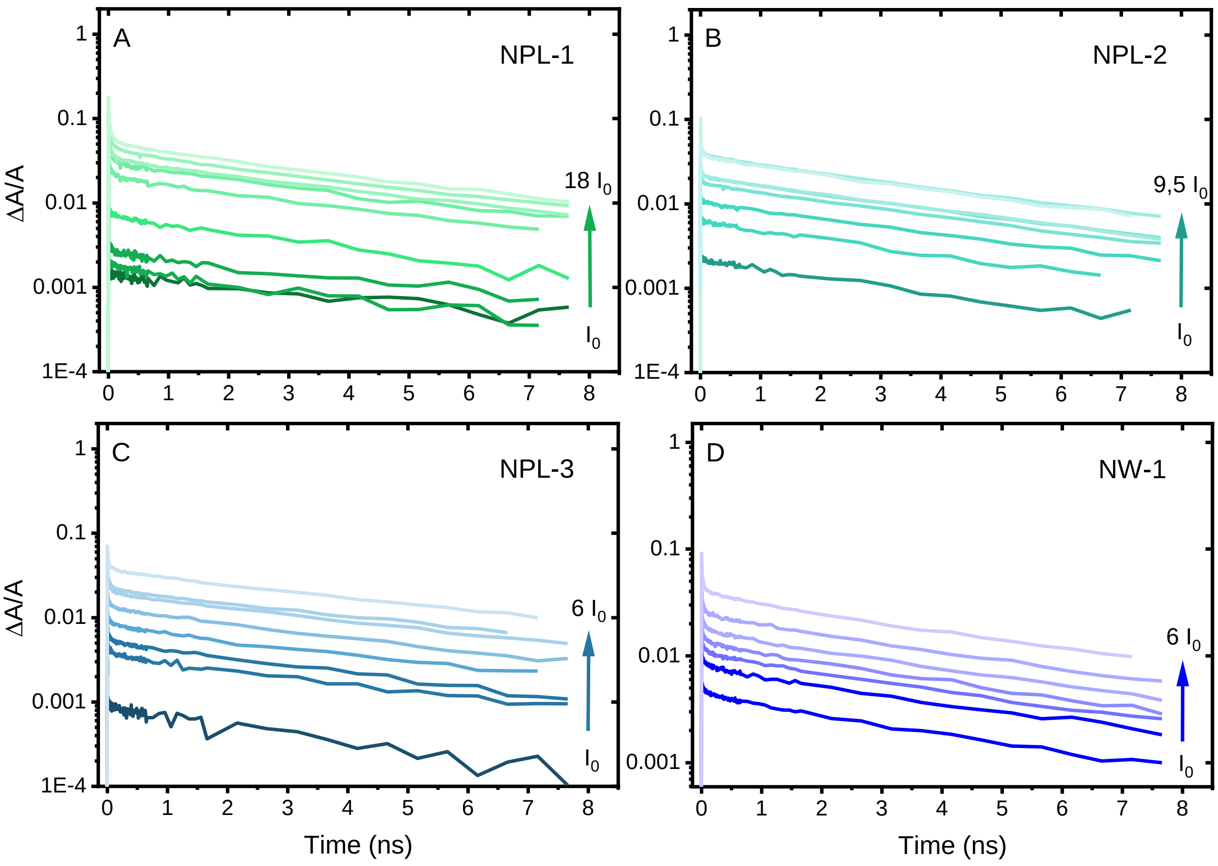

Figure S5 Pump-intensity dependence of TA dynamics for $CsPbBr_3$ NPLs, and NW, pumped at 800 nm.

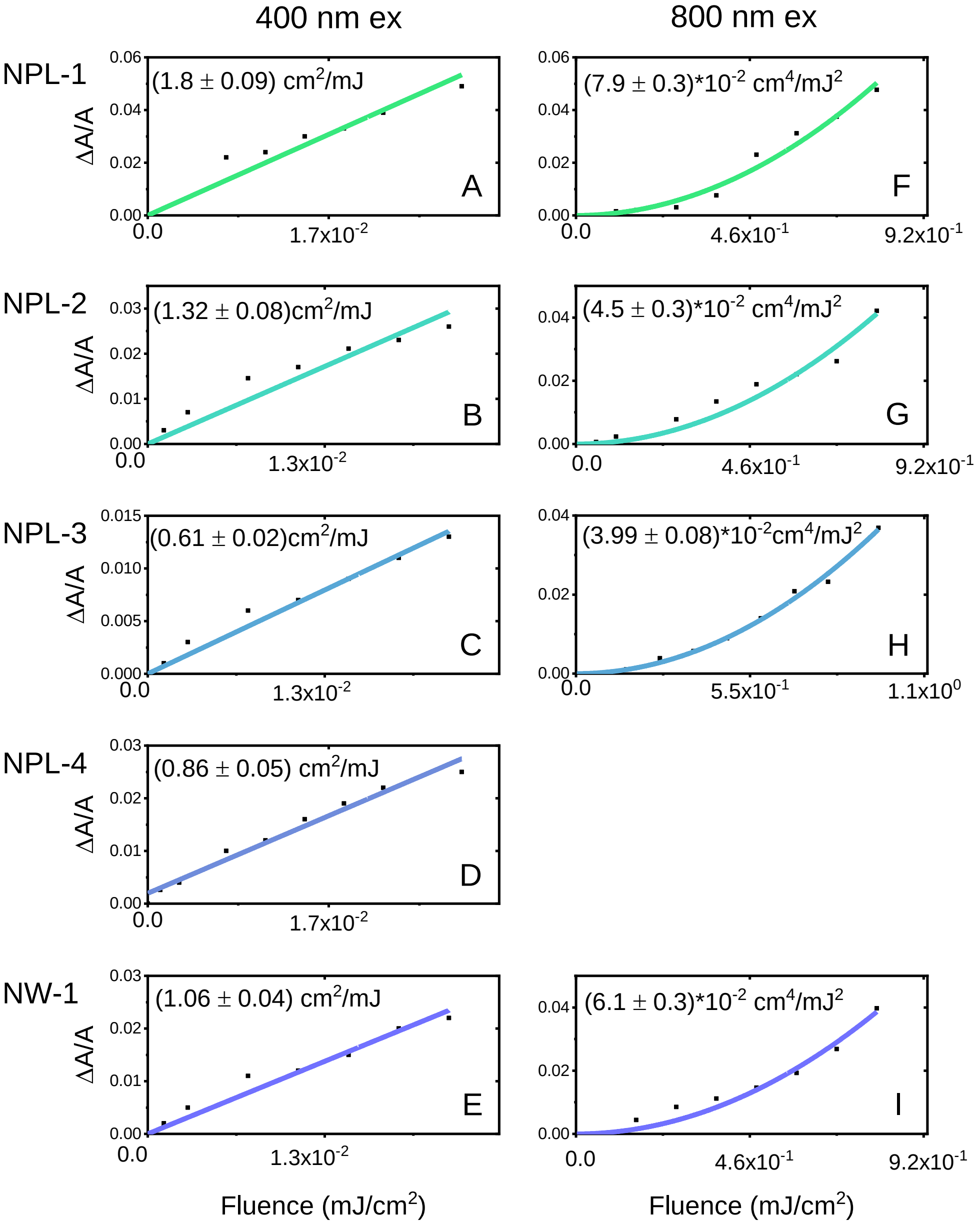

Figure S6. Excitation fluence dependence to ground state bleaching signal (extrapolated to t=0) normalized with absorbance at first exciton transition energy (-ΔA/A) for $CsPbBr_3$ NCs with different dimensions. Left panel: 400 nm excitation; Right panel: 800 nm excitation.

Table S3. Summary of factors C1 C2

Sample	C1 (cm^2/mJ)	C2 (cm^4/mJ^2)
NPL1	1.81 ± 0.09	(7.9 ± 0.3)*10^{-2}
NPL2	1.32 ± 0.08	(4.5 ± 0.3)*10^{-2}
NPL3	0.61± 0.02	(3.99 ± 0.08)*10^{-2}
NPL4	0.86± 0.05	
NW-1	1.06± 0.04	(6.1 ± 0.3)*10^{-2}

The OPLA and TPA induced excitation density (ρ) is related to OPLA and TPA coefficients at weak excitation fluence (the average number of excitons per NC $\langle N \rangle < 1$):

$$\rho = \frac{\int_{-\infty}^{+\infty} I_{400}(t)dt\left(1-e^{-\alpha^1 L}\right)}{hv_{400}} = \frac{I_{400}^{peak}\tau\left(1-e^{-\alpha^1 L}\right)}{hv_{400}} \quad \text{(S9)}$$

$$\rho = \frac{\int_{-\infty}^{+\infty} (I_{800}(t))^2 dt\; \alpha^2 L}{2hv_{800}} = \frac{(I_{400}^{peak})^2 \tau \alpha^2 L}{2\sqrt{2}hv_{800}} \quad \text{(S10)}$$

I_{400}(t) is the intensity of the 400 nm laser pulse, and I_{800}(t) is the intensity of the 800 nm laser pulse, $\alpha^1$is the OPLA coefficient, α^2 is the TPA coefficient, L is the sample thickness (1 mm), v_{400} and v_{800} is the frequency of the 400 nm laser pulse and 800 nm respectively, h is plank constant. Factor 2 in Eq.S4 considers the requirement of two photons to generate one exciton. We assume the 400 nm and 800 nm pulses are Gaussian. Hence, we can carry the integration and express it via the peak intensity (I_{400}^{peak}, I_{800}^{peak}) at 400 nm and 800 nm respectively, and pulse duration (τ=150 fs). With the equations S7 to S10, we can correlate the OPLA and TPA coefficients to the experimentally fitted parameters C_1 and C_2 found in table S3:

$$C_1 \propto \frac{(1-e^{-\alpha^1 L})}{hv_{400}} \quad \text{(S11)}$$

$$C_2 \propto \frac{\alpha^2 L}{2\sqrt{2}hv_{800}\tau} \quad \text{(S12)}$$

We have assumed that the intensity change of the 800 nm laser beam due to the TPA while passing the sample is negligible. Then the TPA coefficient can be calculated as:

$$\alpha^2 = \frac{C_2}{C_1}\frac{\sqrt{2}\tau}{L}(1 - e^{-\alpha^1 L}) \quad \text{(S13)}$$

(S7) TPA Cross-section calculation

Then the TPA cross-section can be calculated from

$$\sigma^2 = \frac{h v_{800} \alpha^2}{(f_\omega)^4 N} \quad \text{(S14)}$$

Where *h* plank constant, *v* frequency at 800 nm, N concentration in particles per cm^3 and $(f_\omega)^4$the corresponding local field factor to the aspect ratio of each sample. The local field factor is explained in the next section. Lastly, we calculate the TPA cross-section divided by the NC volume, a factor mostly known as the TPA coefficient β.

$$\frac{\sigma^{(2)}}{V_{NC}} = \beta \quad \text{(S15)}$$

(S8) Local field Calculation

As mentioned in the main article, geometry is a determinant factor that modulates the local field. The case of a sphere is shown in the main article. As previously said, the sphere is the model used to describe the local field of a cube shape. For NW, the best geometry to describe its dielectric response is the prolate spheroid and for NPL is an oblate spheroid. In Fig S7, the NC structure corresponding to its model for local field calculation is shown.

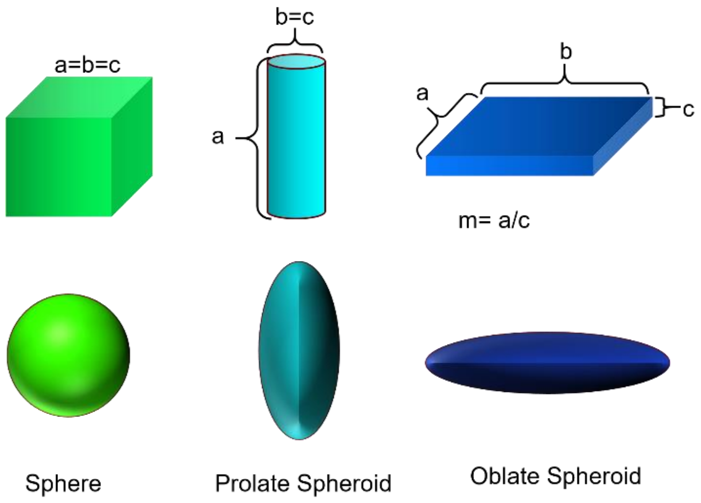

Figure S7 Nanostructure and its corresponding model for local field factor.

We will now discuss the calculation of the local field factor for the oblate and prolate spheroids. For a prolate and oblate spheroid the local field factor is:[10]

$$f_{(w)} = \frac{1}{1+L_i\left(\frac{\epsilon_s}{\epsilon_m}-1\right)} \quad \text{(S16)}$$

ϵ_m= dielectric constant of the surrounding medium (toluene 2.38) and ϵ_s=dielectric constant of the semiconductor $CsPbBr_3$ (7.3).[11] L_i are the depolarization factors L_x, L_y, and L_z. . Osborn[12] has previously calculated these factors.

For a prolate spheroid a >b=c , AR=m=a/c. the a, b, c coordinates are as seen in figure S7

$$L_z = \frac{1}{m^2-1}\left(\frac{m}{2\sqrt{(m^2-1)}} * ln\left(\frac{m+\sqrt{m^2+1}}{m-\sqrt{m^2-1}}\right) - 1\right) \quad \text{(S17)}$$

$$L_{x/y} = \frac{m}{2(m^2-1)}\left(m - \frac{1}{2\sqrt{(m^2-1)}} * ln\left(\frac{m+\sqrt{m^2+1}}{m-\sqrt{m^2-1}}\right)\right) \quad \text{(S18)}$$

And for an oblate spheroid a = b >c, AR=m=a/c

$$L_z = \frac{m^2}{m^2-1}\left(1 - \frac{m1}{\sqrt{(m^2-1)}}\, arcsin\left(\frac{\sqrt{m^2+1}}{m}\right)\right) \quad \text{(S19)}$$

$$L_{x/y} = \frac{1}{2(m^2-1)}\left(m^2\sqrt{(m^2-1)}\, arcsin\left(\frac{\sqrt{m^2+1}}{m} - 1\right)\right) \quad \text{(S20)}$$

(S9) Exciton and multiexciton lifetime.

With the exponential decay fitting of the GSB-decay, the exciton lifetime can be extracted at 400 nm (OPLA) and 800 nm (TPA) excitation.

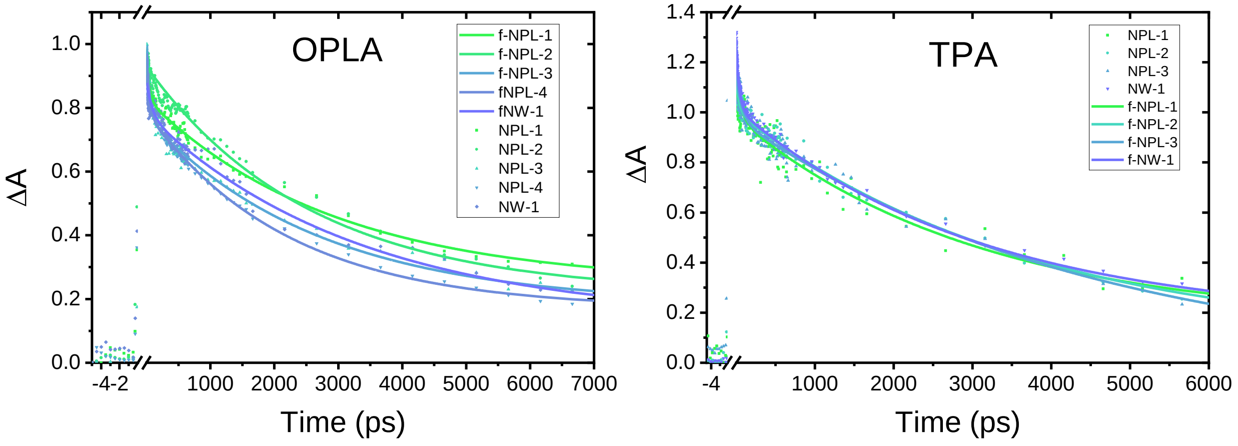

Figure S8. A) GSB decay at 400 nm excitation (OPLA) B) GSB decay with 800 nm excitation (TPA).

Table S4. Summary of calculated exciton and bi-exciton lifetimes at 400 nm excitation and 800 nm excitation.

Sample	Exciton lifetime		Multiexciton lifetime	
	Excitation wavelength			
	400 nm	800 nm	400 nm	800 nm
NPL-1	2404 ± 292	2714 ± 936	13.2± 2.4	11.2 ±1.4
NPL-2	3811 ± 585	3272 ± 573	10.3 ± 2.3	9.1 ± 1.3
NPL-3	2034 ± 225	3629 ± 1346		12.5 ± 2.2
NPL-4	1705 ± 95		9.2 ± 2.7	
NW-1	2901 ± 750	2457 ± 205	7.8 ± 0.1	12.5 ± 2.0
Average	2571 ± 471	3018 ± 293	10.1 ± 1.3	11.3 ± 0.9

(S10) Multiexciton multiplicity.

According to a Poisson distribution, the probability of exciton generation, $P_{<N>^1}$ Exciton and biexciton generation $P_{<N>^2}$ can be expressed as:

$$P_{<N>^1} = 1 - e^{-\sigma I} \quad \text{(S21)}$$

$$P_{<N>^2} = 1 - e^{-\sigma I} - \sigma I e^{-\sigma I} \quad \text{(S22)}$$

Where σ = cross-section and I is the fluence of the excitation pump. Since the ground state bleach intensity in TA kinetics is proportional to the population of excitons or biexcitons generated in the NCs directly after excitation, the amplitudes of the different decay components in TA kinetics decay fitting can be expressed by the above equations according to the exciton species (i.e. exciton, biexciton, etc.) as reported elsewhere.[13].

Since we have already subtracted the single exciton decay component of all the fluence dependent TA kintics in Fig. 4. The amplitude of the residual decay components can actually be used to identify the multiexcitonic species modeled by the above equations. Figure 9 ploted the fluence dependent amplitude of the residual TA bleach decays components after substracting single exciton decay for each sample, together with the exciton (solid lines) and biexciton model (dashed lines).

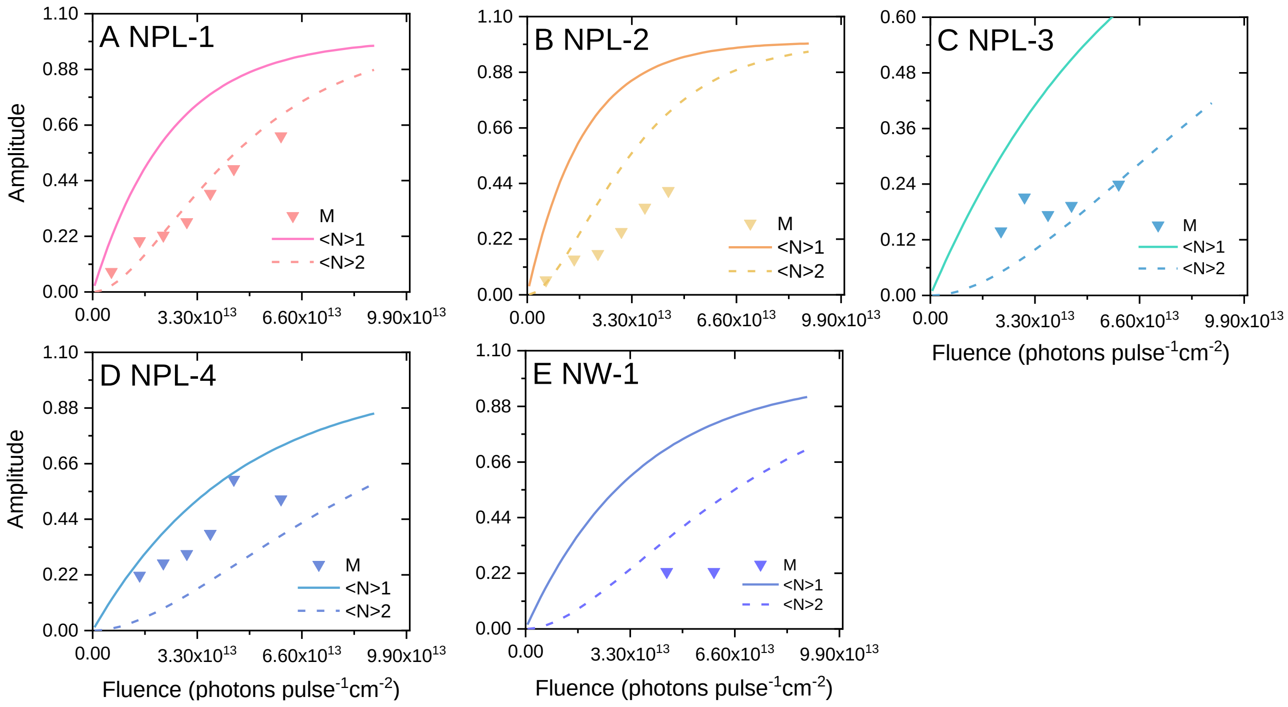

Figure S9 Amplitude of residual TA bleach decay component vesus excitation fluence. The curves are caculated Poisson distribution model for the population of exciton solid line and biexciton dotted line.

(S11) Photo thermal effect discussion

We estimate the local temperature in NPs as follows, reported previously[14]:

Assuming that all the excessive energy of the photon absorbed by NPs has been transformed into heat, the overall photo-induced thermal energy E would be:

$E = \Delta E\langle N\rangle$ = (0.29-1.07) eV (1)

where ΔE is the excess energy of each absorbed photon= (energy of excitation 400 nm) – Bandgap = 3.1 eV-(2.66-2.84)=(0.26-0.44) eV, and $\langle N\rangle$ = (0.95-2.54) per QD per pulse is the mean number of the photons absorbed by one NP at the excitation fluence.

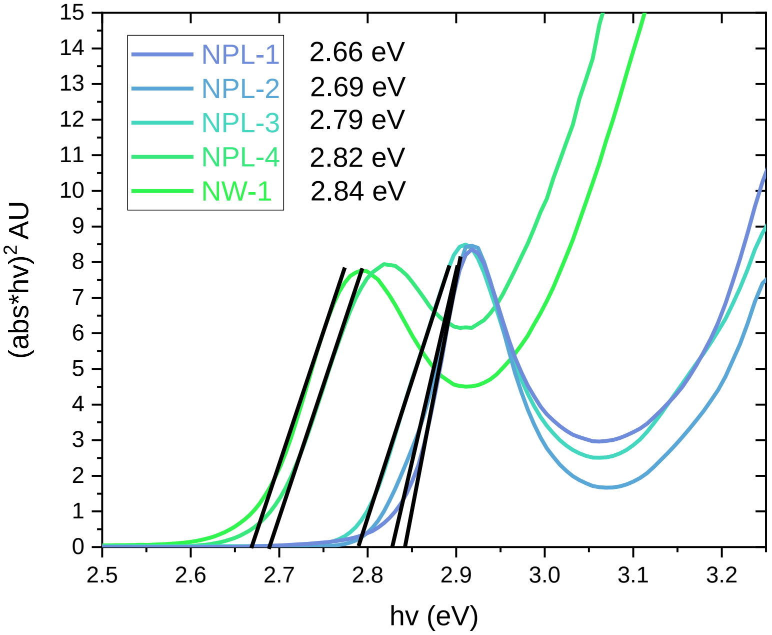

Figure S10. TAUC Plot and determination of the optical bandgap for all NCs.

Here, we restrict the considerations to the heat capacity of a solid state of N atoms. The inner energy consists of the kinetic and potential energy of the atoms. Since all the Pb and Br and Cs atoms have three degrees of freedom, the energy is given by:

$$E = (3N_{Pb\&Br\&Cs})k_B\Delta T = \Delta E\langle N\rangle \qquad (2)$$

where $N_{Pb\&Br\&Cs}$, the number of all atoms in a NP is equal to (5732-11009), kB is the Boltzmann's constant and ΔT is the increase in temperature. Using the E value given in equation 1, we can calculate the expected elevated temperature of the NCs in this case to be about ($1.71\text{x}10^{-5}$ to $5.95\text{x}10^{-6}$) K. The change in temperature is then insignificant.

Table S5. Photo thermal effect parameters and calculation of the increase in temperature from pu excitation.

Sample	# atoms per NC	bandgap eV	ΔE (eV)	<N> max	E (eV)	ΔT (K)
**NPL-1**	7254.20	2.66	0.44	2.43	1.07	1.71E-05
**NPL-2**	11009.79	2.69	0.41	2.54	1.04	1.1E-05
**NPL-3**	5732.21	2.79	0.31	0.95	0.29	5.96E-06
**NPL-4**	6325.19	2.82	0.28	1.31	0.37	6.73E-06
**NW-1**	6601.92	2.84	0.26	1.66	0.43	7.59E-06

(S12) Comparison of β for different $CsPbBr_3$ by Z-scan method

Table S6. Comparison of different reported values of $CsPbBr_3$ from bulk to NCs nonlinear optic properties measured by different z-scan methods.

Sample	*Z-Scan Conditions*	*β (cm/GW)*	*Excitation Wavelength (Nm)*	*Ref*	*Dimensions*
Single Crystal		3.7	800	[15]	
bulk Single Crystal	Wavelength dependent 30 ps, 50 Hz	5	1000	[16]	
Nanocube	396 fs, 1kHz	0.032	787	[17]	(21.4 nm)^3
QD		0.097		[18]	(9 nm)^3
	50 fs, 1kHz	0.038	600	[19]	(12 nm)^3
	50 fs, 1kHz	0.072	700		(12 nm)^3
	50 fs, 1kHz	1.8	800		(12 nm)^3
	100fs, 1kHz	2790	720	[20]	NR [a]
	fs, 1kHz	0.091	800	[21]	
	100fs, 1kHz	0.085	800	[22]	(11.4nm)^3
Nanorod	50 fs, 1kHz	0.005	600	[19]	d=80 nm l=μm scale
	50 fs, 1kHz	0.005	700		
	50 fs, 1kHz	0.0071	800		

Nanoplatelet	70fs, 1kHz	3.9	800	[23]	l=20 nm<br>t= NR
Nanosheet	80fs, 1kHz	10.94	800	[24]	t= 104.6 nm<br>l= NR
	80fs, 1kHz	9.17	800		t=127.2 nm<br>l= NR
	80fs, 1kHz	8.53	800		t=151.5 nm<br>l= NR
	80fs, 1kHz	8.03	800		t=164.6 nm<br>l= NR
	80fs, 1kHz	5.56	800		t= 182.3<br>l= NR
	80fs, 1kHz	4.73	800		t= 195.4<br>l= NR

t= Thickness, length= l, diameter =d, NR= Not reported

[a]= synthesis method melt-quenching-annealing (usually broad size distribution)

The dimensions for most Nanosheets and nanorods have not been determined making it impossible to calculate the AR. From the articles is inferred that the dimensions are in the μm scale. If we estimate them to be 1 μm, we can obtain the following relationship.

The values for the Nanorod do not show the expected trend but we see the same qualitative behavior of nanosheets (assymetric NCs) to have a higher value than the symmetric QDs.

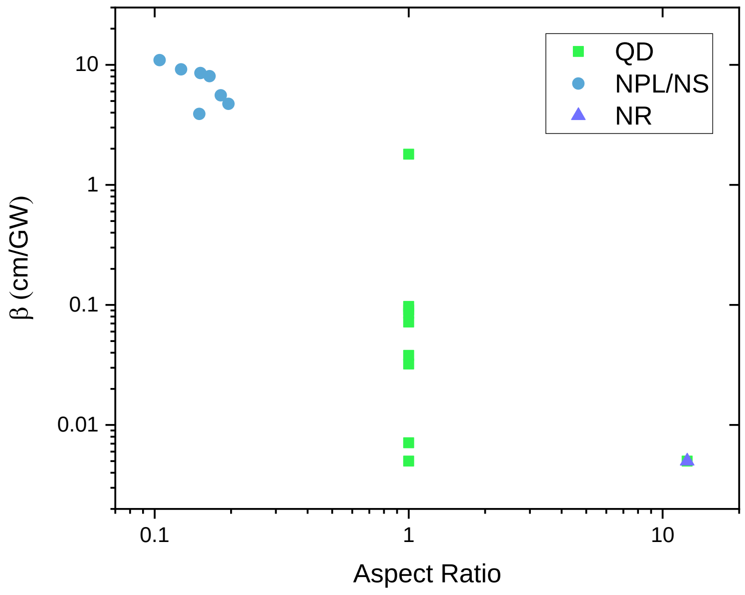

Figure S11. Comparison of β values of CsPbBr3 measured by Z-scan method at 800 nm excitation. QD= Quantum Dots, NPL=nanoplatelets, NS=Nanosheet, NR=nanorods.

(1) Dutta, A.; Dutta, S. K.; Das Adhikari, S.; Pradhan, N. Tuning the Size of CsPbBr3 Nanocrystals: All at One Constant Temperature. *ACS Energy Lett.* **2018**, *3*, 329–334.

(2) Chen, Z.; Yu, C.; Shum, K.; Wang, J. J.; Pfenninger, W.; Vockic, N.; Midgley, J.; Kenney, J. T. Photoluminescence Study of Polycrystalline CsSnI3 Thin Films : Determination of Exciton Binding Energy. *J. Lumin.* **2012**, *132* (2), 345–349.

(3) Zheng, K.; Zhu, Q.; Abdellah, M.; Messing, M. E.; Zhang, W.; Generalov, A.; Niu, Y.; Ribaud, L.; Canton, S. E.; Pullerits, T. Exciton Binding Energy and the Nature of Emissive States in Organometal Halide Perovskites. *J. Phys. Chem. Lett.* **2015**, *6* (15), 2969–2975.

(4) Chen, J.; Žídek, K.; Chábera, P.; Liu, D.; Cheng, P.; Nuuttila, L.; Al-Marri, M. J.; Lehtivuori, H.; Messing, M. E.; Han, K.; Zheng, K.; Pullerits, T. Size-And Wavelength-Dependent Two-Photon Absorption Cross-Section of CsPbBr3 Perovskite Quantum Dots. *J. Phys. Chem. Lett.* **2017**, *8* (10), 2316–2321.

(5) Zhang, F.; Liu, Y.; Wei, S.; Chen, J.; Zhou, Y.; He, R.; Pullerits, T.; Zheng, K. Microscopic Morphology Independence in Linear Absorption Cross-Section of CsPbBr3 Nanocrystals. *Sci. China Mater*. **2021**, *64*, 1418–1426.

(6) Makarov, N. S.; Guo, S.; Isaienko, O.; Liu, W.; Robel, I.; Klimov, V. I. Spectral and Dynamical Properties of Single Excitons, Biexcitons, and Trions in Cesium-Lead-Halide Perovskite Quantum Dots. *Nano Lett.* **2016**, *16* (4), 2349–2362.

(7) Lenngren, N.; Garting, T.; Zheng, K.; Abdellah, M.; Yartsev, A. Multiexciton Absorption Cross Sections of CdSe Quantum Dots Determined by Ultrafast Spectroscopy. *J. Phys. Chem. Lett.* **2013**, *4*, 3330–3336.

(8) Dakovski, G. L.; Shan, J. Size Dependence of Two-Photon Absorption in Semiconductor Quantum Dots. *J. Appl. Phys.* **2013**, *114* (May 2013), 014301.

(9) Dakovski, G. L.; Shan, J. Size Dependence of Two-Photon Absorption in Semiconductor Quantum Dots. *J. Appl. Phys.* **2013**, *114* (1), 1–6.

(10) Achtstein, A. W.; Hennig, J.; Prudnikau, A.; Artemyev, M. V; Woggon, U. Linear and Two-Photon Absorption in Zero- and One-Dimensional CdS Nanocrystals: Influence of Size and Shape. *J. Phys. Chem. C* **2013**, *117*, 25756–25760.

(11) Yang, Z.; Surrente, A.; Galkowski, K.; Miyata, A.; Portugall, O.; Sutton, R. J.; Haghighirad, A. A.; Snaith, H. J.; Maude, D. K.; Plochocka, P.; Nicholas, R. J. Impact of the Halide Cage on the Electronic Properties of Fully Inorganic Cesium Lead Halide Perovskites. *ACS Energy Lett.* **2017**, No. 2, 1621–1627.

(12) Osborn, J. A. Demagnetizing Factors of the General Ellipsoid. *Physical Review*. 1945, pp 351–357.

(13) Yarita, N.; Tahara, H.; Ihara, T.; Kawawaki, T.; Sato, R.; Saruyama, M.; Teranishi, T.; Kanemitsu, Y. Dynamics of Charged Excitons and Biexcitons in CsPbBr3 Perovskite Nanocrystals Revealed by Femtosecond Transient-Absorption and Single-Dot Luminescence Spectroscopy. *J. Phys. Chem. Lett.* **2017**, *8* (7), 1413–1418.

(14) Zheng, K.; Abdellah, M.; Zhu, Q.; Kong, Q.; Jennings, G.; Kurtz, C. A.; Messing, M. E.; Niu, Y.; Gosztola, D. J.; Al-Marri, M. J.; Zhang, X.; Pullerits, T.; Canton, S. E. Direct Experimental Evidence for Photoinduced Strong-Coupling Polarons in Organolead Halide Perovskite Nanoparticles. *J. Phys. Chem. Lett.* **2016**, *7* (22), 4535–4539.

(15) Song, J.; Cui, Q.; Li, J.; Xu, J.; Wang, Y.; Xu, L.; Xue, J.; Dong, Y.; Tian, T.; Sun, H.; Zeng, H. Ultralarge All-Inorganic Perovskite Bulk Single Crystal for High-Performance Visible–Infrared Dual-Modal Photodetectors. *Adv. Opt. Mater.* **2017**, *5* (12), 1–8.

(16) Saouma, F. O.; Stoumpos, C. C.; Kanatzidis, M. G.; Kim, Y. S.; Jang, J. I. Multiphoton Absorption Order of CsPbBr3 as Determined by Wavelength-Dependent Nonlinear Optical Spectroscopy. *J. Phys. Chem. Lett.* **2017**, *8* (19), 4912–4917.

(17) Liu, S.; Chen, G.; Huang, Y.; Lin, S.; Zhang, Y.; He, M.; Xiang, W.; Liang, X. Tunable Fluorescence and Optical Nonlinearities of All Inorganic Colloidal Cesium Lead Halide Perovskite Nanocrystals. *J. Alloys Compd.* **2017**, *724*, 889–896.

(18) Wang, Y.; Li, X.; Zhao, X.; Xiao, L.; Zeng, H.; Sun, H. Nonlinear Absorption and Low-Threshold Multiphoton Pumped Stimulated Emission from All-Inorganic Perovskite Nanocrystals. *Nano Lett.* **2016**, *16* (1), 448–453.

(19) Krishnakanth, K. N.; Seth, S.; Samanta, A.; Rao, S. V. Broadband Femtosecond Nonlinear Optical Properties of CsPbBr_3 Perovskite Nanocrystals. *Opt. Lett.* **2018**, *43* (3), 603.

(20) Zhu, C.; Wang, L.; Zhao, D.; Yang, Y.; Liu, X.; Xu, B.; Xu, Z.; Qiu, J. Multiphoton Upconversion and Non-Resonant Optical Nonlinearity in Perovskite Quantum Dot Doped Glasses. *Opt. Lett.* **2021**, *46* (20), 5216.

(21) Cao, Z.; Lv, B.; Zhang, H.; Lv, Y.; Zhang, C.; Zhou, Y.; Wang, X.; Xiao, M. Two-Photon Excited

Photoluminescence of Single Perovskite Nanocrystals. *J. Chem. Phys.* **2019**, *151* (15).

(22) Wei, K.; Xu, Z.; Chen, R.; Zheng, X.; Cheng, X.; Jiang, T. Temperature-Dependent Excitonic Photoluminescence Excited by Two-Photon Absorption in Perovskite CsPbBr_3 Quantum Dots. *Opt. Lett.* **2016**, *41* (16), 3821.

(23) Ketavath, R.; Katturi, N. K.; Ghugal, S. G.; Kolli, H. K.; Swetha, T.; Soma, V. R.; Murali, B. Deciphering the Ultrafast Nonlinear Optical Properties and Dynamics of Pristine and Ni-Doped CsPbBr3 Colloidal Two-Dimensional Nanocrystals. *J. Phys. Chem. Lett.* **2019**, *10* (18), 5577–5584.

(24) Zhang, J.; Jiang, T.; Zheng, X.; Shen, C.; Cheng, X. Thickness-Dependent Nonlinear Optical Properties of CsPbBr3 Perovskite Nanosheets. *Opt. Lett.* **2017**, *42* (17), 3371.
